# Psychiatric readmissions and their association with environmental and health system characteristics: a systematic review of the literature

**DOI:** 10.1186/s12888-016-1099-8

**Published:** 2016-11-07

**Authors:** Jorid Kalseth, Eva Lassemo, Kristian Wahlbeck, Peija Haaramo, Jon Magnussen

**Affiliations:** 1 SINTEF Technology and Society, Health Research, P.O. Box 4760 Sluppen, NO-7465 Trondheim, Norway; 2 National Institute for Health and Welfare (THL), Mental Health Unit, P.O. Box 30, FI-00271 Helsinki, Finland; 3 Department of Public Health and General Practice, Norwegian University of Science and Technology, Faculty of Medicine, P.O. Box 8905, MTFS, NO-7491 Trondheim, Norway

**Keywords:** Psychiatry, Readmission, Rehospitalisation, Recidivism, Health care system, Environment, Systematic review

## Abstract

**Background:**

Psychiatric readmissions have been studied at length. However, knowledge about how environmental and health system characteristics affect readmission rates is scarce. This paper systemically reviews and discusses the impact of health and social systems as well as environmental characteristics for readmission after discharge from inpatient care for patients with a psychiatric diagnosis.

**Methods:**

Comprehensive literature searches were conducted in the electronic bibliographic databases Ovid Medline, PsycINFO, ProQuest Health Management and OpenGrey. In addition, Google Scholar was utilised. Relevant publications published between January 1990 and June 2014 were included. No restrictions regarding language or publication status were imposed. A qualitative synthesis of the included studies was performed. Variables describing system and environmental characteristics were grouped into three groups: those capturing regulation, financing system and governance; those capturing capacity, organisation and structure; and those capturing environmental variables.

**Results:**

Of the 734 unique articles identified in the original search, 35 were included in the study. There is a limited number of studies on psychiatric readmissions and their association with environmental and health system characteristics. Even though the review reveals an extensive list of characteristics studied, most characteristics appear in a very limited number of articles. The most frequently studied characteristics are related to location (local area, district/region/country). In most cases area differences were found, providing strong indication that the risk of readmission not only relates to patient characteristics but also to system and/or environmental factors that vary between areas. The literature also points in the direction of a negative association of institutional length of stay and community aftercare with readmission for psychiatric patients.

**Conclusion:**

This review shows that analyses of system level variables are scarce. Furthermore they differ with respect to purpose, choice of system characteristics and the way these characteristics are measured. The lack of studies looking at the relationship between readmissions and provider payment models is striking. Without the link to provider payment models and other health system characteristics related to regulation, financing system and governance structure it becomes more difficult to draw policy implications from these analyses.

**Electronic supplementary material:**

The online version of this article (doi:10.1186/s12888-016-1099-8) contains supplementary material, which is available to authorized users.

## Background

Repeated hospitalisations for patients with psychiatric disorders may reflect the type of illness, but also environmental factors and underlying inefficiencies in pre- and post-discharge treatment [1]. Readmissions may be disruptive to patients and their families, and represent a strain on limited health care resources. Readmissions may be avoided by providing adequate treatment during the index hospital stay, combined with an adequate discharge and transition plan and a follow-up regime that allows the patient to remain in the community after discharge. Thus, the probability for readmission will depend on individual characteristics of the patient, but also on factors in the patient’s environment, on the way services are delivered, and on the organisation, governance and financing of health care.

Readmission rates are used as an indicator of quality of hospital care [2, 3], and several strategies have been developed to reduce readmission rates [4]. These include enhanced patient education, more post-discharge follow-up care, and increased coordination with outpatient providers [5]. There is considerable variation in rates of 30-day unplanned readmissions between different high-level income countries [6]. The readmission rate for schizophrenia varied from 5 to 20 % among 20 countries reporting data for the year 2011.

Previous reviews of factors that affect the probability of a patient being readmitted to psychiatric inpatient care indicate that studies of the impact of health care system are scarce. Also, previous reviews have typically identified and discussed system characteristics as pre- or post-discharge service use of psychiatric patients [1, 7, 8]. However, in many instances there is not a clear cut separation between patient characteristics and systems characteristics. Service use as a patient level predictor (pre- or post-discharge) is likely to reflect case-mix characteristics as well as, and sometimes rather than, system characteristics. For instance, length of index hospitalisation may be an indicator of patient needs due to severity of illness as well as reflecting system level differences in capacity, structure or treatment. Likewise, number of outpatient follow-up visits may reflect patients’ needs and also be a system characteristic. Patient level predictors may confound or mask system effects. For example, outpatient follow-up may be associated with an increased likelihood of readmission when measured at patient level (follow-up visits are an indicator of severity), whereas as a system characteristic availability of aftercare may be associated with a reduced likelihood of readmission (preventive measure). To identify system effects using variables characterising service use at patient level, such as length of stay or receiving aftercare visits, will be particularly difficult in analysis of readmission risks based on data for patients treated within broadly speaking the same health care system [9]. Variables measured at a higher than patient level on the other hand, capture effects of characteristics of health care system and other context factors on readmission risks.

European countries differ in how health care is regulated, financed, governed, organised and delivered. Thus, across Europe we observe a multitude of different financial, organisational and institutional mental health care models [6, 10]. Furthermore, local environmental or context factors vary, which may influence both the “demand”- and “supply” side in health care delivery. For policy makers, knowledge about the relationship between environmental and system characteristics and the probability for readmissions is of value. Hence a review of aggregate system characteristics is warranted.

The objective of the present study was to systematically review what type of aggregate system level and environmental characteristics have been studied and examine their association with readmission after discharge from psychiatric in-patient hospital care.

## Methods

The Comparative Effectiveness Research on Psychiatric Hospitalisation by Record Linkage of Large Administrative Data Sets (CEPHOS-LINK) is a European research project investigating psychiatric services across six countries, namely Austria, Finland, Italy, Norway, Romania, and Slovenia, carried out from 2014 to 2017. CEPHOS-LINK aims to compare different types of health service interventions in terms of differences in readmission outcomes in adult patients, who have been discharged from hospital with a psychiatric diagnosis. This will be achieved utilizing state-of-the-art approaches using large ‘real world’ electronic databases, allowing for specific comparisons and detailed analysis to be made. The present study belongs to a series of systematic reviews from the CEPHOS-LINK project on predictors of readmission after discharge from psychiatric or general in-patient health care for patients with a psychiatric diagnosis. The first steps in the literature search process and the initial screening for eligibility were performed in collaboration between participants in the CEPHOS-LINK project.

### Searches

Studies of predictors of psychiatric readmission were identified through comprehensive literature searches conducted in the electronic bibliographic databases Ovid Medline, PsycINFO, ProQuest Health Management, OpenGrey and Google Scholar. Additionally, the reference lists of all included articles were screened for additional papers to be included. Searches were performed using combinations of terms (used as MeSH terms or free text, depending on the database) describing mental health services and readmission. For more detailed description of the search terms, please see Additional file 1.

### Types of studies included

Studies on readmissions after discharge from psychiatric in-patient treatment that include environmental and/or health care system variables were included. To separate patient level pre-discharge and post-discharge variables from the system/environmental variables, and to be eligible for inclusion in the present study, the variables should, be measured at an aggregate level i.e. above patient level, and should be characteristics not of the patient, but of the provider or environment of the patient. There are grey zones between aggregate system and/or environmental variables and patient characteristics, for example when system level characteristics are measured at the patient level. In such cases the studies were included. Relevant publications from January 1990 through June 2014 were included. No diagnostic, time frame, language or publication status restrictions were applied. Only quantitative study designs were included (observational and interventional studies). Studies not covering the issue of readmission were excluded. Admissions to day hospitals or community programs were not considered as readmissions. Studies only reported in abstract format with no access to full text (e.g. conference abstracts) were excluded. Papers not including original data, such as editorials, letters to the Editor, commentaries, reviews and meta-analyses were excluded.

### Types of participants

Only studies examining adult populations (mean age ≥ 18 years) were included in the review. Only studies on patient populations having been admitted to in-patient health care were included.

### Selection of studies

Studies were initially selected for inclusion by two pairs of researchers independently [EL and VD, LS and RS]. Conflicting decisions were resolved by discussion. Full-texts were screened, if necessary, to establish eligibility. Next, all candidate studies were screened once more for eligibility by two researchers [EL, JM]. Full texts of potentially relevant studies were assessed for eligibility by at least two of the authors [EL, JK, JM]. Discrepancies were resolved by discussion by all three authors, until consensus on inclusion, or exclusion, was reached.

### Data extraction

Available structured data on variables associated with readmission were extracted from the included studies, and entered into an evidence evaluation table by two pairs of researchers [EL, JM; EL, JK]. Key characteristics of the studies selected for the systematic review are presented in Additional file 2. A qualitative synthesis of the included studies was performed.

### Environmental and system characteristics

Environmental and system level variables can be defined and operationalised in different ways. For clarity, we make distinction between the following types of variables:

The first category of readmission predictors describes *regulation, financing system, and governance structures.* These variables describe features of the healthcare system along dimensions such as legal and other types of regulation, funding (e.g. tax vs. social insurance), provider payment systems, degree of decentralisation, the extent of financial integration between hospitals and primary (community) care providers, etc.

The second category of readmission predictors describes the *capacity, organisation and structure* of the healthcare providers. Furthermore, they may in general terms describe the composition and structure of the delivery of health and social services within geographical areas (region or community) or within specific provider types (hospital or community providers). Capacity variables can be measured directly in the form of resources (spending, bed rates, staffing rates) or indirectly (e.g. institutional length of stay). Hospital and/or community treatment policies, processes and procedures, treatment orientation and philosophy can be viewed as organisational variables. These may also describe more narrow aspects of the organisation of services, and thus characterize specific interventions. Typical structural variables are size (of hospital or community services), the scope of services provided within a specific setting (e.g. teaching and research in addition to patient treatment), and the case-mix of the hospital. Also variables capturing aftercare are included in this category.

The third category capture what can be denoted as *environmental* variables, i.e. variables that describe the surroundings of the patient. Environmental variables are measured at community level and can be further classified into geographical variables, including variables describing the geographical distribution of population and service (such as area of location of hospital or of residence of patients, population density etc.); demographics (composition of population in terms of age, gender etc.); and socio-economic variables (community income, education level, unemployment rates etc).

## Results

**Fig. 1:**
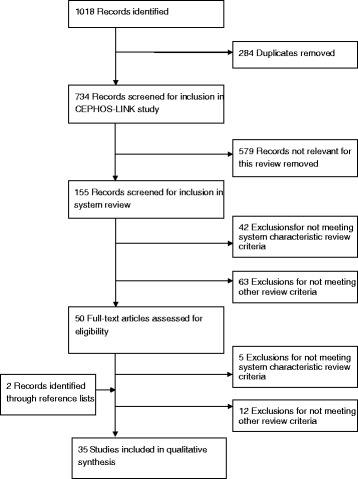
The article selection: CEPHOS-LINK Literature Review Flow Diagram

Comprehensive literature searches returned 1 018 records, of which 734 were unique, the process is depicted in the flow-chart of Fig. 1. Of the 734 unique records identified in the original CEPHOS-LINK search, 155 were identified as potentially describing system level or environmental readmission predictors. Following additional screening, 50 articles were included for full text assessment of eligibility. Review of full texts left 33 articles for inclusion. Upon checking the references of these, two more were added. Finally, 35 articles were included in the literature review. An overview of the included articles is provided in Additional file 2.

### Type and level of analysis

Eight of the included articles were studies of interventions. Of these, three were randomized controlled trials and five were case-control type studies. Five studies could be characterised as “natural experiments” and 22 were other types of observational studies. Of the latter, six used aggregated readmission rates either at the program/hospital level, or small area (administrative) level. The remaining 16 observational studies used patient level data, and (in all but one) multivariate analytical methods, i.e. controlling for possible confounders. The follow-up period included both short (up to 30 days), medium (30 + days-1 year), and long (>1 year) follow-up times. The interpretation of results differs for patient level analyses and analyses of aggregated readmission rates at hospital or area level. Patient level analyses separate patient level (“need”) effects and “pure” system or environmental level effects. In aggregated readmission rates analyses, the results will comprise both aggregated patient level effects and system/environmental effects.

The system and environmental characteristics identified in each study are summarised in Additional file 2. Characteristics are put in one of the three broad categories for environmental and/or system characteristics described above, and also according to level of measurement: physician level, hospital level and community level.

### Regulation, financing system, and governance structures

Three studies investigated the impact of system characteristics falling within this category [11–13]. The studies are heterogeneous both in terms of system (Israel vs. US), and in terms of variables studied (legislation, carve-out and utilisation management programs respectively). Grinshpoon et al. [11] found that passing of rehabilitation legislation in Israel aiming at establishing infrastructure in the community for the rehabilitation of patients with severe mental disorders was associated with increased survival in the community. The two US studies looked at different aspects of health insurance policies targeted towards cost-containment and/or quality improvement likely to affect outcomes in mental health care. Utilisation Management programs potentially restrict access to services and treatment by imposing preadmission certification, concurrent review, and case management. The program was found, by limiting length of stay, to increase the risk for early readmission for psychiatric patients [13]. Behavioural health carve-outs means that mental health and substance abuse benefits are separated from general medical benefits. Carve-out was not found to affect risk of readmission for major depressive disorder [12]. Although these studies illustrate the possible relevance of aggregate level system characteristics, it is difficult to draw conclusions based on isolated studies.

### Capacity, organization and structure

The majority of the studies (69 %) included in this review falls into this category. They can further be divided into studies capturing potential *hospital size effects* [14–17], *capacity variables* [18–23], *resource availability/utilisation/ quality* [14, 17, 24, 25], *length of stay* [14, 16–18, 20], *case-mix* [14, 20], *treatment policies or orientation* [14, 16, 17, 26], *hospital type* [24, 25, 27, 28] and *community aftercare* [14, 16, 17, 29–36].

*Hospital size* is measured differently across studies (e.g. number of beds, staff, patient volume). With the exception of a study by Lee and Lin [15] which found a positive association between patient volume and readmission rates, none of the studies were able to establish any association between size and readmission rates.

*Capacity* is also captured through a number of different variables, i.e. beds -, staff -, spending (hospital and/ or community) -, or patient volume *per capita*. Again, in the majority of studies (4 out of 6) there was no association between capacity and readmission rates. There are however, some exceptions. Øiesvold et al. [18] found differences in time to readmission between areas with different bed rates, with areas with an intermediate level of beds per population associated with the highest readmission risk. Wan and Ozcan [20] found both spending at area level and per capita patient volume to be positively associated with higher readmission rates, however Turner and Wan [19] did not find any association between readmission rates and mental health spending per population.

*Resource availability/utilisation/quality* can be captured by staff-patient ratios and staff composition. Although results are conflicting, Heggestad [24] found that accessibility to therapists was negatively associated with readmission rates, and Lin and Lee [25] found that patients treated by psychiatrists with the highest caseloads had the highest likelihood of being readmitted. Others [14, 17] did not find any association between overall staff-patient ratio and readmission rates, suggesting that availability of specialist may be more important than overall staffing ratios.

Hospital *length of stay* is consistently found to be negatively related to (risk/rate of) readmission when measured both at hospital level or community level [14, 16–18, 20]. Heggestad [24] also found that high patient turnover (annual discharges/beds) increased the likelihood of readmissions.

Characteristics capturing institutional *case-mix* were found to be associated with readmission rates in studies at aggregated small area or program level [14, 20]. However, case-mix may vary across several dimensions, i.e. diagnosis or form of admission (voluntary, compulsory), and it is difficult to draw conclusion from the results of these limited analyses.

Characteristics capturing *treatment policies, process/procedure and treatment orientation* are viewed as system variables by way of not being patient level (individual characteristics) variables. However as treatment policies as well as procedures vary, these studies are difficult to summarize. As an example Moos et al. [17] and Peterson et al. [14] report correlations/associations between program level readmission rates and different treatment process and/or orientation variables for patients with substance abuse disorders. However, the recent large scale patient level study of Mark et al. [16] did not find any effect of hospital-level procedure variables when controlling for other influential variables.

Niehaus et al. [26] studied the effect of the implementation of the policy of early (“crisis”) discharge as a response to pressure on bed capacity. The results showed a far higher risk of readmission for patients discharged due to bed pressure than patients discharged as usual.

Some studies included variables describing *hospital type*. There is, however, no common international typology of hospital types, and again the results of these studies are likely to be context specific. Studies that include hospital type have looked at degree of specialisation, ownership and type of hospital wards. A series of studies from Taiwan reported associations with degree of specialisation [25, 27, 28] and hospital ownership [25, 27]. Public hospitals were found to have higher readmission rates than private for-profit and non-profit hospitals. Heggestad [24] did not find differences in risk of readmission between patients discharged from wards in general hospitals and psychiatric hospitals.

Several studies include the type and extent of *community aftercare.* Generally, the results depend on the type of care, but suggest overall that providing aftercare follow-up visits [14, 16, 17], or designing specific aftercare programmes in the communities [29–32, 35], may reduce readmission rates.

### Environment

Studies including environmental variables comprise *geographical characteristics* [12, 13, 21, 23, 25, 27, 28, 37–44], *demographic composition* [19–21, 23], and *socioeconomic variables* [19–21, 23, 44, 45].

*Geographical* characteristics such as population and service *location* were included in several studies. The most commonly used – and the simplest –are area variables or area comparisons [12, 13, 23, 25, 27, 28, 37–42]. In the majority of these studies (10 out of 12) significant area differences in readmission rates were found. Studies including area/region/country variables, or comparing different areas, may capture system level differences related both to regulation, financing and governance, capacity, organisation and structure, as well as environmental factors, but do not directly investigate specific system variables. However, some studies that include area level variables provide an explicit motivation/discussion that relates to health care system differences. Sytema [38, 39] compares readmission between areas at a different stage of deinstitutionalisation by way of comparing Victoria (Australia) and Groningen (The Netherlands) [38] and also including Verona (Italy) [39]. Differences are interpreted as being the result of capacity and/or organisational variables, however without these variables explicitly included in the analysis.

Lower readmission rates in urban regions were found in two studies [21, 28]. Rüesch et al. [21] also found a positive association between readmission rates and population density, i.e. combined, the results of the two variables seem to capture non-linearity in the effect of density. Other studies, however, do not find any association between population density [23, 43] or distance to services [44] and readmission rates.

Variables characterising the *demographic* composition of communities like age composition [20], gender composition [21], ethnicity [19, 20, 23], foreign born population [21, 23] were also identified. High share of foreign born were found to be positively associated with hospital readmissions in both studies. The other variables were not significantly associated with readmission risk/rate in any of the studies.

*Socio-economic characteristics* of the community have also been investigated in the included studies. Three studies included income level. The two studies involving area level readmission rate [20, 21] did not find any effect of community income (measured as median income/average taxable income in the area), while Prince et al. [23] found the risk of readmission to be lower for elderly patients living in high income communities than in low income communities (four categories (quartiles) of median community income). Education level was included in three studies. Stahler et al. [44] found lower likelihood of being readmitted for patients residing in communities with a higher educational attainment. The two studies performed at area level found either an inverse bivariate effect or did not find any effect of this variable [19, 21]. Other socio-economic variables studied were unemployment [19, 45], poverty [19, 20], deprivation and social class level [19, 21, 45], family structure and crowded household [19, 21], and a range of substance abuse specific variables in Stahler et al. [44]. Most often no significant associations with readmission rates were found. The exception being Turner and Wan [19] finding lower readmission rates in areas with the lowest socio-economic status (composite measure of unemployment, crowed household, low education, and poverty) and finding the share of female-headed households to be positively associated with readmission rates.

## Discussion

This review identified 35 studies including one or more system and/or environmental characteristics. The vast majority of studies look at variables characterising capacity, organisation and structure (24 studies), and variables describing the environment (17 studies). Only three studies included characteristics within the category of regulation, financing system and governance structure.

The most frequent type of variable studied was related to location of services or patients (local area, district, region, country). In most cases area differences were found. Hence, there is a strong indication that the risk of readmission not only relates to patient characteristics but also to system and/or environmental factors that varies between areas. However, simply adding location to the analysis will help adjust for factors that differ systematically between geographical areas, but without detailed information about factors such as capacity, governance structures or treatment profiles, and environmental characteristics, there is little policy relevant information from these variables.

A few studies included characteristics measuring the effect of size and capacity of service systems. However, the way these system features were measured varied. Most often no association with readmission rates were found (8 studies out of 11). Some studies, measuring size or capacity by patient volumes or spending levels, found positive association with hospital readmission rates. Patient volumes have been found to be positively associated with readmission for psychiatric patients when measured both at physician, hospital and community level [15, 20, 25]. Both patient volumes and spending may capture other systems features as well as size and capacity. At physician and hospital level, patient volume also captures case-load and patient turnover, and at community level it may capture “demand”. Likewise per capita spending on mental health on an area level may capture service structure; e.g. a hospital based system could be more expensive than community based system [46].

Differences in capacity may also indirectly be captured by other types of variables related to capacity utilisation and patient turnover. Average length of stay was systematically found to be negatively associated with readmission risk/rates. The interpretation of this result typically refers to short stays indicating premature discharge [47]. Depending on the heterogeneity of hospital types included in the study, average length of stay could also capture other factors like different patient populations and hospital functions. However, the more direct test of the early discharge hypothesis provided by the study of the policy of “crisis” discharge in case of bed shortage supported the hypothesis [26]. Likewise, another “natural experiment” aimed at cost containment also showed similar results, i.e. the effect of utilisation management on shortening length of stay contributed to higher risk of readmission [13].

Several studies also investigated the effect of, or included characteristics capturing the effect of, the use of aftercare at system level. Most often positive results were found, i.e. that higher share of patients receiving aftercare, or specific aftercare or community services interventions, contributed to lowering readmission rates.

The review identifies only a few studies investigating the level or availability of community resources and no clear picture on the impact on hospital readmissions emerge. While e.g. the passing of rehabilitation legislation in Israel was associated with reduced likelihood of readmission [11], no differences in readmissions were found for regions in Massachusetts, US, with very different levels of funding for community programs [22]. The policy relevance of such results is difficult to assess without information on other characteristics describing structure, capacity and organisation of the health and social care system.

Environmental characteristics, other than area, have also been investigated. However, the type of characteristics are scattered and most often no significant effects are found.

Conflicting results for environmental factors such as variables capturing socio-economic environment i.e. income, education and other measures of socioeconomic conditions at community level may relate to both how the variables are measured and what they actually capture. Firstly, conflicting results could of course reflect deficiencies in the data and measurement of variables, e.g. if selection of patients or census data are biased in some way. Secondly, different measures capture different contextual aspects of living in socio-economic advantageous/disadvantageous neighbourhoods, and both level and dispersion of community income may matter. Thirdly, conflicting results could reflect variability in the level of measurement. A narrow definition of neighbourhood may fail to capture relevant neighbourhood aspects, while if measured at a very aggregate level the within area variation may be too high and the between area variation too low to capture any contextual effects. Further work is needed to understand the right level of measurement for environmental factors, which could vary according to study objectives and type of variables. Finally, the effect of environmental variables may depend on context. The channels by which the effect of environmental variables operate are complex - from individual response to physical environment and social norms and culture to service accessibility and quality, and may interact with both individual characteristics as well as other environmental or system variables.

Many of the results found in this review are in line with previous reviews. Lien [8] concluded that longer length of stay and follow-up visits after discharge seem to be important determinants of readmission rates. These results typically were based on patient level predictors. In an early review of the literature Klinkenberg and Calsyn [7] found that receipt of aftercare was associated with lower readmission rates. Vigod et al. [48] reviewed the literature on transitional interventions and found that in about half of the included studies the intervention had a statistically significant impact on readmission rates.

The lack of studies looking at the relationship between readmissions and provider payment models is striking. Provider payment models inherently send powerful signals to service providers likely to affect the delivery of care such as patient turnover and length of stay. It is perhaps not so surprising that there are few studies of such health systems characteristics since most studies are single-country analyses and these system characteristics may not vary a lot within a country. Such variables may also be difficult to study in cross-country analysis, other than on a speculative basis, unless the number of countries entering analysis is high enough to control for other confounding system level or environmental variables. Still, it is somewhat surprising that we were able to identify only four studies involving patient level cross-country comparisons of predictors of hospital readmission for patients with a psychiatric diagnosis.

The (possible) effects of regulation, financing system and governance structure on readmission rates are likely to go through how the financing, organisation, structure and capacity of the delivery system are affected, and this will be captured by studies that include such variables. Again the type and measurement of variables included varies. It is also surprising that so few studies include size and capacity measured by number of beds and bed rates. Whether this indicates that information on hospital beds is difficult to collect, either due to lack of data or to restrictions on access to information on service provider identity, or lack of interest in these types of variables, is hard to tell. With hospital or catchment area identification in patient level data, several types of aggregate system type variables can be constructed, such as average length of stay and patient case-mix, as well as different type of environmental variables (geographical, demographical and socio-economic factors), that typically are easily accessible, and could thus be investigated. It is therefore also surprising that so few studies include case-mix variables to control for differences in type of hospital, and also that so few studies have investigated environmental variables such as distance to nearest inpatient service and other factors likely to affect service use and aggregate service needs. A caution is also warranted since system level variables need to be carefully interpreted. Observed differences in readmission rates related to e.g. hospital ownership could capture other system characteristics such as payment methods, as well as other correlated factors such as unobserved differences in patient case-mix, type and accessibility of community after-care etc.

We have included studies that analyse the effects of specific models of care in a community setting. The policy relevance of local interventions critically depend on how well they are defined and described as well as whether they can be applied outside of their specific context. The main policy implication from these studies is that the way care is delivered matters. This is hardly surprising, but serves to underline that while there is a growing literature that discusses models of care, we still lack knowledge of the institutional factors that promote good care.

This review provides a systematic attempt to take into account all literature addressing the impact of system and environmental variables on hospital readmissions within mental health, covering publications over a more than 20 year period and providing an extensive and systemised coverage of different system and environmental characteristics. Limitations of this systematic review should also be mentioned. The included studies vary a lot both in terms of the outcome variable (the length of time to follow-up after discharge), the patient population studied, number of observations, the type of analysis and methods used, level of analysis, and the type and number of variables included both at patient level and system level. Hence, it is not possible to draw strong conclusions on the impact of specific variables.

The interpretation of effects for system level and environmental variables crucially depend both on level of analysis and the control for related factors. The strength of this review is that it explicitly addresses health system and environmental factors measured as aggregate level variables. Analysing the impact of health system characteristics using individual level variables involves the possibility of misinterpreting results, confusing the effect of patient level service use on readmission risk as an expression of health system effects where it may capture different patient needs (service use as signal of severity of illness). Likewise, the effects of aggregate level variables (e.g. community education level) do not apply directly at the individual level since the aggregate variable often measures a different (group or context) property than the namesake at the individual level. System or environmental variables studied in analysis of aggregate readmission rates could capture both (aggregate of) patient needs and system level effects. Hence, the ecological inference fallacy may go both ways [49]. Using patient level data and controlling for same type of variables both at patient and system (hospital and/or community) level in a multi-level design enables separation of effects.

## Conclusions

This review identifies gaps in the literature on hospital readmissions for patients with psychiatric diagnoses. The included studies provide strong indication of the importance of health system and other context factors. However, studies of system level variables are limited in numbers and they differ both with respect to purpose, choice of system characteristics and the way these characteristics are measured. While a number of studies include variables that relate to capacity, organisation and structure, it becomes more difficult to draw policy implications from these analyses without the link to provider payment models and other health system characteristics related to regulation, financing system and governance structure. Since such variables may not vary much within a country, cross-country comparative analyses might contribute to the understanding of the impact of system characteristics and why we observe differences between countries in readmission rates for patients with psychiatric diagnoses. Understanding the link between health system and environmental variables on the one hand, and hospital readmissions on the other is important for policy development as well as for planning and optimising mental health service delivery.

## Additional files

Additional file 1: Detailed search strategies. (DOC 47 kb)
Additional file 2: Characteristics and main results of the studies included in the review. (DOCX 53 kb)
