# Supplementary material for: Psychiatric readmissions and their association with environmental and health system characteristics: a systematic review of the literature
Source: BMC Psychiatry. 2016 Nov 7;16:376. doi: 10.1186/s12888-016-1099-8 (PMC5100223; doi:10.1186/s12888-016-1099-8)
Supplement: Additional file 2: — Characteristics and main results of the studies included in the review. (DOCX 53 kb) [file 12888_2016_1099_MOESM2_ESM.docx]

Characteristics and main results of the studies included in the systematic review on psychiatric readmissions and their association with environmental and health system characteristics.

| **Author (first), year** | **Study population,**  (incl. country) | **Aim of the study** | **Level of measurement,**  **Type of analysis,**  **Follow-up period**  **(**short**:** ≤30 days,  medium: >30 days & ≤1 year, long: >1 year) | **Environment or system predictor**  RFG=Regulation, Financing system and Governance structure, COS=Capacity, Organisation and Structure, E=Environmental* | **Results**  (sign in parenthesis after variable indicate association with readmission) |
| --- | --- | --- | --- | --- | --- |
| Observational studies | | | | | |
| Eaton, 1992 [37] | First time hospitalised patients with schizophrenia,  N=483-9,663.  Australia, Denmark, England USA | Study of risk for readmission in cohorts of schizophrenic patients in four register areas: Victoria, Australia; Maryland, U.S.A.; Salford, England; and Denmark. | Individual,  Multivariate,  Short term | **E**: Area.  Observes differences across 4 systems (countries). No real analyses of system, merely observations. | **E:** Differences in the risk of readmission between areas observed. |
| Heggestad, 2001 [24] | All diagnoses,  N=5,520.  Norway | To study the association between hospitals' operating conditions and the risk of early readmission. | Individual,  Multivariate,  Short term | **COS**: *Hospital* - patient turnover (annual discharges/beds), bed-occupancy rate (days/ (beds*365)), accessibility of therapists (per inpatient day), type of hospital (general ward vs psychiatric hospital).  *Community* - out-patient consultations/ 1000 pop, GP/ 1000 pop | **COS:** *Hospital* **-** patient turnover (+), bed-occupancy reverse U-shape, access to therapists (–) |
| Hodgson, 2001 [45] | All diagnoses 16-64,  N=3,404.  UK | 1. To examine the effects of clinical, administrative and demographic variables on readmission  2. To develop a prediction model for readmission using Cox regression. | Individual,  Multivariate,  Long term | **E:** MINI deprivation index, unemployment rate and Jarman UPA score. | **E**: No significant associations. |
| Husted, 2000 [43] | Patients with SPMI.  Area level analysis (87 counties).  USA | To investigate if readmission rates correlates with the population density in the areas. | Aggregate (mean readmission rate),  Bivariate,  Medium term | **E:** Population density | **E**: Population density - No correlation. |
| Korkeila, 1998 [41] | All diagnoses,  N=49,455.  Finland | To investigate factors predicting readmission and the interval between readmissions to psychiatric hospitals during the early 1990s in Finland. | Individual,  Multivariate,  Long term | **E:** hospital region | **E**: Differences between regions found |
| Lee, 2007 [15] | Patients with schizophrenia, bipolar disorder, MDD,  N=31,528.  Taiwan | Examines the association between a hospitals’ psychiatric inpatient volume and 30-day readmission rates. | Individual,  Multivariate,  Short term | **COS**: *Hospital*: Hospital volume (Admissions and days). | **COS**: Hospital volume (+). |
| Lin, 2010 [28] | First-time hospitalized psychiatric patients,  N=44,237  Taiwan | To explore the risks and rates of readmission and their predictors 14 days, one year, and five years after discharge for the psychiatric population in Taiwan. | Individual,  Multivariate,  Short, Medium, and Long term | **COS**: *Hospital* -accreditation level  **E**: Hospital location | **COS**: lower accreditation level (+)  **E**: residence in less-urbanized regions (+).  Strongest association with short and medium term readmission. |
| Lin, 2009 [25] | Patients hospitalized for the first time for mental disorders,  N=66,959.  Taiwan | Aimed to compare psychiatrists' in-patient caseload volume with length of stay (LOS) and 30-day readmission rates in Taiwan. | Individual,  Multivariate,  Short term | **COS**: *Psychiatrist* –volume (case load), age and gender. *Hospital* - accreditation level (size and tech clinical service capability), ownership.  **E**: Hospital location. | **COS**: *Psychiatrist* - case load (+), *Hospital* - lower accreditation level (+), Ownership: Public > for profit > non-profit  **E**: differences between locations. |
| Lin, 2006 [27] | Patients with schizophrenia,  N=29,373.  Taiwan | To explore the association between length of stay (LOS) and 30-day readmission rates for hospitalized patients with schizophrenia in Taiwan. | Individual,  Bivariate shown for system variables,  Short | **COS:** *Physician* - age, gender. *Hospital* - ownership, accreditation level, teaching status.  **E**: Hospital location | **COS**: *Physician-*  age (-), *Hospital* – differences according to accreditation level: Regional> district>medical centre and ownership: Public>for profit>non-profit  **E**: differences between locations. |
| Mark, 2013 [16] | Patients with mental and/ or substance use disorder (M/ SUD),  N=121,271.  USA | To provide data on readmissions for M/ SUDs to inform debate over hospital readmission as an actionable quality performance indicator. | Individual,  Multivariate,  Short | **COS**: *Hospital* - Median LOS, the mean annual Medicaid admissions for M/ SUD, and the % of Medicaid admissions with a principal M/ SUD diagnosis that received particular procedures. *After-care* -Post-discharge follow-up patterns that occurred within the first 7 days of discharge. | **COS** (At 1%-level): *Hospital* - median LOS (-),  *After care*: CMHC visit (-). (Outpatient visits and prescription fills (+) at 5 %-level) |
| Moos, 1995 [17] | Substance abuse patients.  Program level analysis (88 programs).  USA | The study compared older substance abuse patients to middle-aged and younger patients treated in 88 specialized substance abuse programs and identified program characteristics associated with casemix-adjusted readmission rates. | Aggregate (readmission rate),  Bivariate,  Medium term | **COS**: *Hospital*: (1) Program structure: total patients, staff/ patient ratio. (2) Policies/ services: Structured Policies, Flexible Discharge Rules, Comprehensive Assessment, Family or Friend Interviews, Family Treatment, Community Consultation, Psycho-educational Emphasis, Group Treatment, Intensity of Treatment. (3) Treatment orientations: Social Skills, Rehabilitation, Therapeutic Community, Family. (4) Program use: Index episode ALOS,  *After care*: Two or more mental health visits during 1 month after discharge (% of patients) | **COS:** *Hospital -* Policies/services: correlation depending on age group. Treatment orientations: (-) for youngest age group. ALOS (-). *After care* - mental health (–) (significant for ages 35+) |
| Peterson, 1994 [14] | Substance abuse patients.  Program level analysis (101 programs).  USA | What program factors are associated with readmission rates after accounting for patient case-mix?  Examine the relative importance of patient vs program factors in explaining variations in program effectiveness. | Aggregate (ratio of actual vs expected readmission rate),  Multivariate,  Medium term | **COS**: *Hospital* – Program structure: staffing (FTE treatment staff, FTE non- psychiatrists physicians (%), FTE certifies addiction counsellors (%), # beds, MSs or PhD level staff or RN (%), Service load, Average staff costs per day. Treatment process: Compulsory admissions (%), Family or friend interviews, Intended treatment duration, Stress management training, Self-help meetings/ week, First week dropout rate (%), Discharged AMA (%), Policy of wait of >= 1 month for readmission.  *After care* - two or more visits in 30 days (%). | **COS:** *Hospital* - % compulsory admissions (-), % family and friend interviews (-), Median days intended treatment duration (-), first week drop-out rate (+), *After care* - visits (-) (latter significant at 10 percent level) |
| Prince, 2008 [23] | Primary psych diagnosis. ICD9- ex dementia or organic disorders 65+,  N=41,839.  USA | Examined predictors of psychiatric readmission among elderly persons. | Individual,  Multivariate,  Medium term | **COS**: *Community*- state MH per capita exp  **E**: Location of facility (type area/ urbanity), region of facility, median community income, % black or Hispanic, % foreign born | **E**: Regional differences, Median income (-), % foreign born (highest)(+) |
| Rüesch, 2000 [21] | All diagnoses.  Area level analysis (171 communities).  Switzerland | While considering variables of the social context of the community as well as of the background of the individual, it tries to take into account both the ecological and the individualistic view of the relationship between social conditions and (treated) mental disorder. | Aggregate (re-admission rate),  Multivariate,  Medium term | **COS**: *Community* - Physicians density.  **E**: Population density, Urban Region, Women, Foreigners, One- person households, social class level, Education, Income | **E**: proportion of foreigners (+), population density (+), and urban region (-). |
| Stahler, 2009 [44] | Dually diagnosed patients,  N=380.  USA | Investigate the role of neighbourhood influences in treatment compliance and continuity of care as well as readmission among dually diagnosed patients following discharge from an acute intensive care hospital unit. | Individual,  Multivariate,  Medium term | **E**: Vacant housing (%), High school diploma (%). Distance of patient’s home from location of discharge hospital, distance (m) to; Bar, Deli beer outlet, AA, NA, Check cashing store, Drug sale arrest density (per km^2^), Drug possession arrest density (per km^2^). | **E**: NA distance(-), High school diploma(-) |
| Sytema, 1999 [38] | Patients with functional non-affective psychosis,  N=1988 (V) and 269 (G).  Australia, Netherlands. | The study compared service consumption, continuity of care and risk of readmission of patients with schizophrenia and related disorders in two systems in a different stage of deinstitutionalization (Victoria, AU and Groningen, NL). | Individual,  Multivariate,  Long term | **E:** Area variable. | No significant differences between systems (effect of area-variable). Separate hazard ratio analysis show different patterns of significant patient level variables in the two systems. |
| Sytema, 2002 [39] | Patients with diagnosis of schizophrenia and related disorders,  N= 31,601.  Australia, Italy, The Netherlands | To test: (1) the length of stay is shorter in a community-based system than in a hospital-based system; and (2) the risk of readmission is independent of the characteristics of the mental health system. Comparing systems in a different stage of deinstitutionalization (Victoria, AU, Groningen, NL, Verona, IT). | Individual,  Multivariate,  Long term | **E**: Area variable. | **E:** Significantly lower risk of readmission in Victoria. Separate hazard ratio show different patterns of significant patient level variables in the three systems. |
| Thornicroft, 1992 [42] | Long stay psychiatric patients,  N=357.  England | To identify risk factors which increase the likelihood of readmission for long stay psychiatric patients after discharge from hospital. | Individual,  Multivariate,  Long term | **COS**: *Hospital*-which hospital, | **COS:** *Hospital* - Differences between hospitals observed in univariate analysis. |
| Turner, 1993 [19] | Chronically mentally ill patients.  Area level analysis (six years for each of the 95 counties and six cities).  USA | Examined the impact of community characteristics on readmission of chronically mentally ill (CMI) clients from public psychiatric hospitals in Virginia. | Aggregate (% readmitted),  Bivariate/Multivariate,  n/a | **COS**: *Community* - #CMIpatients/1000pop, #all psych inpatients/1000pop, % psychiatrist/1000pop, acute psych bed/1000pop, non-psych MDs/10000pop, $/capita MH services.  **E**:% civilian unemployment pop, % households > 1/person per room, % pop not completing high school, % pop below poverty level, % black pop, %female headed households | **E**: female-headed households (+), socioeconomic status (low status (-)) |
| Wan, 1991 [20] | Patients in state (VA) facilities. Area level analysis (four years for each of the forty CSBs).  USA | To develop an analytic framework and methodology for estimating the demand for psychiatric hospitalisation in state facilities, either new admissions or readmissions of clients from the CSBs of Virginia. | Aggregate (Number of readmissions per 1,000 CSB population per year),  Multivariate  n/a | **COS**: *Community* - Case-mix in state facilities ( % black, % male, % chronically ill); # new patients/1000 pop, Average length of stay (ALOS), #beds/1000, Number of services offered, MH$/capita;  **E**: %65+/1000, %black, %poor, median income. | **COS**: *Community* - % chronically mentally ill patients in state facilities (+), # new patients/1000 pop (+), ALOS (-), Number of services offered (+), MH$/capita (+) |
| Zilber, 2011 [40] | All diagnoses,  N=6,868.  Israel | To develop a predictive model for 30-day readmission, examining specifically the effect of the length of the inpatient stay preceding the discharge. | Individual,  Multivariate,  Short term | **E:** Area variables. The six districts differ in the availability of community services (measured by no mental health agents per 10,000 adult pop). | **E**: TelAviv +Center(+) |
| Øiesvold, 2000 [18] | All new patient admitted to the psychiatric services in study,  N=837.  Denmark, Finland, Norway, Sweden | (i) Do patient characteristics and factors related to utilization of psych services predict readmission risk?  (ii) Does the relationship between these factors and readmission risk show a more common pattern across gender, diagnostic group and sector?  (iii) Do availability and accessibility of psych health care resources predict readmission risk? | Individual,  Multivariate,  Medium term | **COS**: *Hospital* - medianLOS, Number of beds/1000 pop, staff/1000 pop (could not be analysed simultaniously) | Sectors with highest median LOS have the lowest readmission risk, no clear pattern for bed size (lowest and highest rates lowest readmission risk). Analysing number of staff in the same way as number of beds gave the same result as the strong correlation between total number of beds and staff |
| Natural experiments | | | | | |
| Fisher, 1992 [22] | All diagnoses,  N=624-1,732.  USA | Examined the proposition that a revolving state hospital door is an inevitable consequence of deinstitutionalisation and that enhancing resources for community based care can limit this phenomenon. | Individual,  Bivariate,  Long term | **COS**: Comparing community tenure in region with high level of resources for community based care (due to federal court decree) (Region I) with other regions. | **COS**: Region I not different from other regions. |
| Grinshpoon, 2007 [11] | Patients with schizophrenia,  N=4,160.  Israel | The objective of the present study was to evaluate the impact of rehabilitation legislation on the readmission of schizophrenic patients. | Individual,  Multivariate,  Long term | **RFG**: Implementation of RMDA - rehabilitation of the mentally disabled act. Comparisons pre- and post-passing of the law. | **RFG**: Implementation of RMDA (-) for schizophrenic  patients with an in-patient stay longer than 6 months  (chronic patients) |
| Merrick, 1999 [12] | Patients with major depressive disorder (MDD),  N=310.  USA | To analyse the effects of the 1993 Massachusetts behavioural health carve-out for state employees on readmissions and follow-up treatment after hospitalisation for MDD. | Individual,  Multivariate,  Long term | **RFG:** Comparisons of pre- and post passing of carve-out  **E**: Region | **RFG:** Carve-out **-** No significant effect |
| Niehaus, 2008 [26] | Male acute psychotic patients aged 18-60,  N=438.  South Africa | Severe pressures on beds in psychiatric services have led to the implementation of an early ("crisis") discharge policy in the Western Cape, South Africa. The study examined the effects of the crisis discharge policy on readmission rates. | Individual,  Multivariate,  Long term | **COS:** Crisis discharge. | **COS**: Crisis discharge (+) |
| Wickizer, 1998 [13] | Patients with a psychiatric diagnosis,  N= 2,443.  USA | To determine whether treatment restrictions imposed on privately insured psychiatric patients by a utilization management program affected the likelihood of readmission. | Individual,  Multivariate,  Medium term | **RFG:** Reduction in length of stay (LOS) resulting from Utilization management (UM) program  **E**: Region | **RFG**: Reduction in length of stay (LOS) resulting from Utilization management (UM) program (+)  **E:** Regional differences |
| Intervention studies | | | | | |
| Gillis, 1990 [29] | Black psychiatric patients,  N=51.  South Africa | A controlled investigation to identify which patients gain particular benefit from a controlled standard home-visiting procedure, and to evaluate cost-effectiveness. | Individual,  Case-crossover combined with case-control study,  Medium term | **COS:** Home-visiting procedure by community nurses. | **COS:** Home-visiting procedure by community nurses (-) |
| Kolbasovsky, 2009 [32] | Patients with primary psychiatric diagnosis, N=652.  USA | The purpose of this study is to determine the impact of Intensive case management (ICM) on 30-day inpatient psychiatric recidivism and associated costs among adult health plan members at elevated risk of psychiatric hospitalisation. | Individual,  Intent-to-treat case control study,  Short term | **COS:** An intent-to-treat, historical control, of intensive case management (ICM) | **COS**: ICM (-) |
| Papageorgiou, 2002 [36] | Compulsory admitted patients,  N=156.  England | To evaluate whether use of advance directives by patients with mental illness leads to lower rates of compulsory readmission to hospital. | Individual,  Randomised controlled  trial study,  Medium term | **COS:** The trial compared usual psychiatric care with usual care plus the completion of an advance directive. | **COS: A**dvance directive – No significant effect |
| Patterson, 1998 [33] | SPMI,  N=196.  USA | The purpose of this study was to evaluate the efficacy of an ICM program in extending community tenure for a cohort of SPMI outpatients served by a CMHC. | Individual,  Case-control study,  Long term | **COS:** Compared the outcomes across a number of indexes for clients receiving ICM services relative to a sample of SPMI outpatients receiving less intensive care. | **COS**: ICM (+) |
| Rössler, 1995 [34] | Schizophrenic patients,  N=2*97.  Germany | To compare a group of patients in the aftercare of case management services (case-managed group) with a pairwise matched control who received no outpatient care from case management services after discharge (control group). | Individual,  Matched case control study,  Long term | **COS:** Compared a group of schizophrenic patients in the aftercare of case management services with a group of patients who received no outpatient care by case management services after discharge from hospital. | **COS**: Case management – No significant effect |
| Reynolds, 2004 [30] | All diagnostic groups except dementia,  N=25.  Scotland | To test a transitional discharge model designed to assist patients discharged from acute admission wards to adjust to community living. | Individual,  Randomised controlled  trial study,  Medium term | **COS:** An intervention called transitional discharge. | **COS**: Transitional discharge (-) . NB Small N, large 95%-CI |
| Schmidt-Kraepelin, 2009 [35] | Patients suffering by schizophrenia or schizoaffective disorder,  N=93.  Germany | To prevent readmissions and thus to optimize satisfaction with treatment and quality of life in patients suffering by schizophrenia or schizoaffective disorder. | Individual,  Matched case-control study,  Medium term | **COS:** A complex intervention (Integrated care program) with improved cooperation between in- and out- patient services. | **COS**: Integrated care program (-) |
| Tomita, 2014 [31] | Patients with psychotic disorder,  N=150.  USA | Assesses whether Critical Time Intervention (CTI) improves the quality of family relationships between family members and individuals living with SMI, and examines whether changes in quality of family relationship mediated the association between the intervention and psychiatric readmission outcomes. | Individual,  Randomised controlled  trial study,  Long term | **COS:** A randomized controlled trial that assessed the effect of CTI in preventing homelessness. | **COS**: CTI (-) |

* Other abbreviations used: AA=Alcoholics Anonymous, AMA=Against medical advice, ALOS=Average Length of Stay, CBS= Community Services Boards, CMHC=Community Mental Health Centres, CMI= chronic mental illness , CTI=Critical Time Intervention, FTE=Full time equivalent, GP= General practitioners, ICM=Intensive case management, MD=Medical doctor, MDD=Major Depressive disorders, MH=Mental health, MS= Master of Science , M/SUD=Mental and/or substance use disorders, NA= Narcotics Anonymous, PhD= Doctor of Philosophy, pop=Population, RMDA=Rehabilitation of the Mentally Disabled Act, RN= Registered nurses, SPMI= Severe and Persistent Mental Illness.
